# Translucency and Masking Ability of Various Composite Resins at Different Thicknesses

**Published:** 2014-09

**Authors:** Farideh Darabi, Golpar Radafshar, Maryam Tavangar, Reza Davaloo, Aref Khosravian, Nastaran Mirfarhadi

**Affiliations:** a Oromaxillofacial Developmental Disease Research Center, Dept. of Operative Dentistry, School of Dentistry, Guilan University of Medical Sciences, Rasht, Iran.; b Oromaxillofacial Developmental Disease Research Center, Dept. of Periodontology, School of Dentistry, Guilan University of Medical Sciences, Rasht, Iran.; c­­ Dentist, Private Practice, Tehran, Iran.; d MS in Nursing Education, Oromaxillofacial Developmental Disease Research Center, School of Dentistry, Guilan University of Medical Sciences, Rasht, Iran.

**Keywords:** Translucency, Composite resins, Masking, Thickness

## Abstract

**Statement of the Problem:** Optical properties of the composite resins, concerning their translucency and thickness, are affected by discolored tooth structure or inherent darkness of the oral cavity.

**Purpose:** This study aimed to compare the translucency parameter (TP) of five different composite resins in different thicknesses and to evaluate their masking ability in black backgrounds.

**Materials and Method:** Five brands of composite resins; Gradia (GC) and Crystalline (Confi-dental) in opaque A2 (OA2), Vit-l-escence (Ultradent) in opaque snow (OS), Herculite XRV (Kerr) and Opallis (FGM) in dentin A2 (DA2) shades were selected to enroll the study. Color coordinates of each composite were determined at 0.5, 1, and 1.5 mm thicknesses on a white backing, the backing of material itself and a black backing were calculated by using a spectrophotometer to evaluate the translucency parameter (TP) of the study materials. The masking ability was also calculated from the specimens on the material itself and on black backing. The values under 2 were estimated as imperceptible. One-way ANOVA, T-test and Tukey HSD were employed for statistical analysis.

**Results:** The masking ability values, recorded for the 1.5 mm-thick specimens, were in the range of imperceptible except for the Herculite. There was no difference in TP values of the materials at 1.5 mm thickness. Opaque snow shade of Vit-l-escence and opaque A2 shade of Gradia showed lower TP values in comparison with the other 1 and 0.5 mm-thick materials and this difference was statistically significant (p< 0.05).

**Conclusion:** In relatively thin thicknesses (≤1mm), these opaque/dentin shade composite resins could not mask the black background color.

## Introduction

The inherent translucency of resin composites can set off some difficulties in shade matching, particularly in severely discolored tooth structures or through-and-through class III and IV restorations [1-3]. In such situations, composite resin restorations can result in a grayish shade or poor-color match up since the translucent materials are affected by the discolored tooth structures or even the darkness of the oral cavity [4-6]. To minimize the effect of background color, shade composite resins (dentin/opaque) have been used as a backing in a layering technique [7-9].The proper knowledge of differences in translucency and the required thickness to mask dark background of the applied resin seems to be essential, though little information is available [4-5, 10-11].

Kamishima et al. [12] evaluated the translucency of two resin composites in different thicknesses (0.5, 1.0, 2.0, 3.0 and 4.0 mm) and found that regardless of shade, the translucency increased exponentially as thickness was decreased. Ikeda et al. [5] evaluated the translucency parameter and masking ability of three resin composites with two shades (A3 and opaque A3) and in 1- and 2- mm thicknesses. They concluded only 2mm thickness of the opaque-shade materials could mask the dark background. Kim et al. [13] evaluated the adequate thickness of six opaque shade composite resins to mask black and gray backgrounds. They reported that a C4 background was masked by resin thicknesses of 0.5-1 mm while a black background required a thickness of 1-2 mm. The optical properties for the shade categories (enamel, dentin, opaque) of the composite resins, produced by different manufactures, are material specific [14]. Therefore, available brand of composite resins in a market of our country should be investigated separately.

The purpose of this study was to compare the translucency parameter of several composite resins in different thicknesses and to evaluate their ability to mask black background color.

## Materials and Method

Five brands of opaque or dentin A2 shade resin composites; Gradia (GC; Tokyo, Japan), Herculite XRV (Kerr; Scafati, Salerno, Italy), Vit-l-escence (Ultradent; South Jordan, USA), Crystalline (Confi-dental; Louisville, USA) and Opallis (FGM, Brazil) were enrolled in this study. Stainless-steel split plates in 0.5, 1, 1.5 mm thicknesses and with a hole of 18 mm in diameter were used as the molds to produce standardized specimens. Each mold was filled with resin composite material and covered with clear celluloid strips on the top and the bottom of the hole. The metal plate was pressed between two glass-slides for 10 seconds, and then the glass slides were removed. The specimens were then light cured for 40 seconds in eight overlapping areas with two light-curing units (Litex 680; Dentamerica, USA) simultaneously. The light intensity was 400m W/cm^2^ and the output of the light was checked with a radiometer. Five specimens from each material thickness were made and after storage in distilled water for 24 hours, the specimens were polished with a wet 1500-grit silicon carbide paper (3M ESPE; St. Paul, USA) on both sides.

The CIE L*a*b*(CIELAB) technique was employed in the present study. This technique is introduced by the International Commission on Illumination (French Commission Internationale de l'éclairage (CIE) which is an organization that establishes the standard values used worldwide to measure color. The values used by CIE are called L*, a* and b* and the color measurement method is called CIE L*a*b* (CIELAB). L* is lightness, where 100 is completely white and 0 is completely black, and a* and b*are red-green and yellow- blue chromatic coordinates, respectively. A positive a* or b* value represents a red or yellow shade respectively [4-8].

In the current study, three backgrounds; white tile (L*=94.32, a*= -0.46, b*=1.26), black tile (L*=0.06, a*= -0.01, b*=0.01), and resin itself were used to determine the translucency parameter (TP) (between black and white backgrounds), and to mimic a black oral cavity (between black and resin backgrounds). 

To determine the CIELAB values of each specimen with each background, color measurements were performed by employing spectrophotometer (Color-Eye 7000 A; Gretag Macbeth, USA).Optical contact was achieved by using an optical fluid (refractive index =1.5) between the composite resin specimen and background. Light source illumination was matched with the average daylight (D65). The translucency parameter of the material at various thicknesses was calculated using the following equation:

$$\text{TP}={\left[{\left({\text{L}}_{\text{w}}^{*}-{\text{L}}_{\text{B}}^{*}\right)}^{2}+{\left({\text{a}}_{\text{w}}^{*}-{\text{a}}_{\text{B}}^{*}\right)}^{2}-{\left(b_{\text{w}}^{*}-b_{\text{B}}^{*}\right)}^{2}\right]}^{\frac{1}{2}}$$

The subscript" W" and "B" refers to the CIELAB values for each specimen on white backing and black backing, respectively. The ability of each material to mask dark oral cavity was determined by calculating the ΔE* of the specimens between the material itself and on black backing using the following equation: 

$$\Delta \text{E}={\left\{{\left(\Delta {\text{L}}^{*}\right)}^{2}+{\left(\Delta {\text{a}}^{*}\right)}^{2}+{\left({\Delta b}^{*}\right)}^{2}\right\}}^{\frac{1}{2}}$$

A smaller ΔE*indicates that the specimen is less sensitive to (as in better able to mask) the black back ground color. The ΔE*value was assessed for each thic-kness and a value of ΔE*≤2 was considered clinically imperceptible regarding the method used by some previous studies [5, 15].

To evaluate any statistical changes in TP of different thicknesses in each composite, one-way analysis of variance (ANOVA) was done. To compare the TP between different materials at the same thickness, Tukey HSD test was performed and it was set at the 0.05 level of significance.

## Results

The median values L*, a* and b* of each composite at three thicknesses and backgrounds and also the values of ΔE* for each material at different thicknesses are indicated in Table 1.

**Table 1:** Mean (SD) of CIE l*, a*, b* and ∆E values in 0.5,1 and 1.5 mm thicknesses of resin composites over backgrounds

**Material**	**Shade**	**Thickness**	l* (SD)			a*(SD)			b*(SD)			**∆E(SD)**
			**White**	**Material** **itself**	**Black**	**White**	**Material** **itself**	**Black**	**White**	**Material** **itself**	**Black**	
Gradia	OA_2_	0.5	67.49(0.12)	67.4(0.12)	64.64(0.18)	2.66(0.13)	2.22(0.73)	2.47(1.22)	14.37(0.4)	14.36(0.4)	11.85(0.34)	4.03(0.29)
		1	69.31(0.5)	68.44(0.41)	66.25(0.2)	4.36(0.19)	4.04(0.19)	3.88(0.27)	16.63(0.35)	15.62(0.62)	14.87(0.37)	2.32(0.3)
		1.5	67.32(0.11)	65.83(0.17)	64.94(0.21)	3.84(0.12)	2.88(0.05)	1.27(0.03)	13.67(0.2)	12.25(0.25)	12.10(0.11)	1.84(0.02)
Herculite	DA_2_	0.5	69.65(0.3)	69.07(0.76)	66.37(0.33)	2.41(0.24)	1.26(0.2)	3.6(0.16)	13.72(0.25)	13.55(0.25)	10.82(0.25)	4.5(0.29)
		1	70.79(.13)	68.72(0.35)	65.45(0.34)	1.98(0.34)	2.56(0.38)	2.85(0.17)	14.52(0.24)	13.48(0.94)	14.22(3.24)	4.6(1.55)
		1.5	69.92(0.79)	69.11(0.31)	68.55(0.32)	4.56(0.06)	3.82(0.08)	2.94(0.09)	8.76(1.82)	9.59(0.14)	7.12(0.09)	2.68(0.04)
Crystalline	OA_2_	0.5	85.37(0.01)	84.67(0.04)	79.95(0.17)	5.31(0.15)	4.39(0.18)	3.23(0.17)	25.33(0.12)	24.96(0.01)	21.68(0.63)	5.87(0.51)
		1	73.13(0.3)	72.09(0.24)	69.63(0.24)	8.48(0.23)	7.03(0.2)	7.83(0.25)	19.27(0.24)	16.17(0.25)	17.05(0.18)	3.36(0.08)
		1.5	55.23(0.2)	54.31(0.3)	52.7690.5)	4.4(0.15)	3.95(0.1)	3.14(0.14)	13.66(0.18)	11.96(0.18)	11.03(0.13)	1.99(0.59)
Vit-l- escence	OS	0.5	83.36(0.1)	84.36(0.11)	80.92(0.11)	1.29(0.16)	1.24(0.15)	1.07(0.08)	13.42(0.09)	12.85(0.05)	11.32(0.09)	3.76(0.01)
		1	80.03(0.49)	79.64(0.31)	78.92(0.44)	0.58(0.37)	1.38(0.41)	1.88(0.4)	12.43(0.43)	11.98(0.43)	8.73(0.46)	2.72(0.02)
		1.5	76.8(0.2)	75.81(0.26)	73.97(0.13)	0.52(0.14)	0.91(0.08)	1.06(0.06)	13.2(6.23)	11.64(5.48)	10.95(0.19)	1.97(7.93)
Opallis	DA_2_	0.5	73.5(0.15)	72.79(0.17)	68.74(0.78)	1.34(0.09)	1.21(0.25)	0.17(0.23)	16.47(0.02)	15.55(0.01)	13.73(0.01)	4.57(0.55)
		1	80.88(0.88)	80.91(0.45)	78.24(0.26)	2.43(0.445)	1.84(0.39)	1.12(0.39)	12.44(0.44)	11.14(0.44)	8.65(0.36)	3.73(0.18)
		1.5	76.97(0.2)	75.69(0.25)	75(0.18)	2.42(0.12)	2.71(0.1)	1.33(0.06)	13.91(0.09)	11.86(0.1)	11.45(0.09)	1.6(0.07)

l*,lightness; a*, redness( positive +a*) or greenness( negative -a*); b*, yellowness(positive + b*) or blueness(negative - b*).

OA2, opaque A2; EA2, Enamel A2; DA2, Dentin A2; OS, Opaque snow

One-way ANOVA test showed significant difference in TP values indifferent thicknesses of each material except for Gradia OA2 shade (*p*< 0.05). Thicknesses with bold ΔE*values are the minimum thicknesses with a ΔE*lower than 2 and indicate the critical thickness required to mask black background.

The ΔE* values recorded for the 1.5 mm-thick specimens were in the range of imperceptible, except for dentin A2 shade of Herculite. All of the composites at 0.5- and 1- mm- thicknesses could not mask the black background. The comparison of the TP of different composite resins with the same thickness is illustrated in Table 2.

**Table 2:** Mean (SD) TP values of different materials

**Material**	**Color**	**TP(SD) Thickness **		
		**0.5**	**1**	**1.5**
Gradia	OA_2_	3.95(0.11)^a^	3.57(0.32)^a^	3.38(0.05)^a^
Herculite	DA_2_	6.09(0.02)^b^	4.53(0.59)^b^	2.96(0.94)^a^
Crystalline	OA_2_	6.66(0.15)^b^	4.7(0.11)^b^	3.82(0.46)^a^
Vit-l-escence	OS	4.07(0.06)^a^	3.65(0.02)^a^	3.22(0.07)^a^
Opallis	DA_2_	5.66(0.57)^b^	4.82(0.4)^b^	3.33(0.07)^a^

In each column the same superscript indicates no significant difference for different material with the same thickness. There was no difference in TP values of opaque/dentin shade of the materials at 1.5 mm thickness. Opaque snow (OS) shade of Vit-l-escence and opaque A2 (OA2) shade of Gradia showed lower TP values in comparison with the other 1 mm and 0.5 mm-thick and it was statistically significant. 

## Discussion

In the present study, the translucency and masking ability of the opaque or dentin color of five different composite resins at three thicknesses were evaluated. These five brands were chosen because of their relative acceptance and popularity by clinicians. We also had to choose the opaque shades of Gradia and Vit-l-escence companies because of the unavailability of dentine shades.

Our results indicates that the alteration of background color from white to material itself and black color leads to a decrease in quantity of the L*, a*, and b* values regardless of the thickness used. This means that all five composite resins, when having up to 1.5 mm thickness, were rather translucent and were influenced by background color.

The ΔE* values of different composites for masking dark background in this study, showed a negative relationship with their thickness. This result is in agreement with previous studies [12-14]. The threshold for clinically acceptable color difference has been reported as ΔE*≤2 [15-16], ΔE*≤3.3 [17], ΔE*≤3.7 [18]. The present study followed the suggestions stated in the Gross et al. [15] study. The study described that the values of ΔE*between 0 and 2 are imperceptible and values of ΔE*in the range of 2 to 3 are just perceptible, 3 to 8 are moderately perceptible and values above 8 are markedly perceptible [15]. According to this classification, none of the studied resins in our study masked the background darkness when used in 0.5-1 mm thickness. However, 4 types of composites (Gradia OA2, Vit-l-escence OS, Crystalline OA2, and Opallis OA2) possessed a ΔE*<2 in 1.5 mm of thickness, hence, they could mask black background in this thickness successfully.

In a recent study, it has been reported the minimum thickness of six opaque shade composite resins (Charisma, Heraeus Kulzer; Estelite Sigma Quick, Tokuyama; Gradia Direct Anterior, GC) was 1.85-2 mm to be able to mask the black background [19]. They also reported that in each composite brand, translucency parameter of OA_3_ shade was higher than OA_2_ shade.

Kim et al. [13] evaluated the translucency and masking ability of six composite resins (Z-350 OA_3_, Amelogen Universal OA_2_, Esthet-X OA_2_, Esthet-X OA_4_, Charmfil UO, and Aelite Universal OA3). They reported none of the investigated opaque resin materials could effectively mask the black background color in 0.5 and 1 mm thicknesses when ΔE*≤2.

Ikeda et al. [14] also evaluated the translucency and masking ability of Fiitek A_110_ (3M), Estelite (Tokuyama Dental) and Unifil S (GC) in two shades (A_3_, OA_3_) and two thicknesses (1 and 2 mm). They reported that only 2-mm-thick opaque shade composite resins could mask the black background color. Although we tested different materials, our result is in line with these findings. DA2 shade of Herculite composite showed a ΔE between 2-3; which based on the classification, it could partially mask the background darkness. This would probably lead to a slightly show-through appearance when finished and polished.

Considering the clinically accepted ΔE* ≤3.3 to mask black backing by several studies [13, 17, 20]; Herculite DA2 in 1.5mm of thickness, Gradia OA2 and Vit-l-escence OS in 1 mm of thickness would successfully mask the background darkness. This finding indicates that the light specifications of the examined resins are noticeably subjective to their thickness.

In this study we also compared the opaque composite resins in the same thicknesses and different thicknesses of the same composite resin. 

With 1.5 mm of thickness, there was no statistically significant difference between TP of composites in any of the materials. But in 0.5 and 1 mm of thickness, TP values for Gradia OA2 and Vit-l-escence OS color were significantly lower than the other materials (*p*< 0.05). Therefore, the materials of choice in this study for using in thin layers could be one of the above mentioned composites.

Because of various clinical conditions, selecting the most appropriate material to be used in all different circumstances appears to be impossible. 

A decrease in the thickness of Gradia OA2 from 1.5 to 0.5 mm did not change the TP values significantly. One possible explanation is the incorporation of micro fine polymerized resin fillers together with silica fillers which are mixed in a concise proportion and size, based on the manufacturer's definitions. This specific structure and composition provides numerous interfaces between fillers and matrix that delivers variable reflecting characteristics and close similarity to the internal reflection properties of natural teeth. 

It is postulated that the color and translucency of esthetic and restorative materials are influenced by filler particles, opacifiers, resin matrix composition, flowability, light curing, and resin polymerization [21-24]. According to Inokishi et al. [20], the more difference between the refraction index of filler and matrix, the better the opacity of resin. This phenomenon is because of the presence of multiple refraction and reflections at filler-matrix interface.

Yu et al. [25] showed that the correlation between TP values and ΔL*was very high. The percent of Bis-GMA also has a significant effect on the translucency of silica filler-containing resin composite [26]. Naeimi et al. [24] compared the effect of different shades (from A to D and C) of two composite resins (Esthet-X and Filtek Supreme) on their translucency and concluded that the translucency of the body shades of Filtek Supreme and the enamel shades of Esthet.X decreased significantly towards the C and D shades.

The absence of the samples with thicknesses between 1 and 1.5 mm was one of the limitations of the present study that is recommended to be assessed in future studies. 

## Conclusion

The relatively thin specimens (≤1 mm) could not mask the black background color. However, all specimens could mask it in 1.5 mm of thickness except for Herculite composite resin.
